# Meeting the Multifaceted Needs of Expectant and Parenting Young Families Through the Pregnancy Assistance Fund

**DOI:** 10.1007/s10995-020-02922-6

**Published:** 2020-05-08

**Authors:** Amy Margolis, Tara Rice, Mousumi Banikya-Leaseburg, Ann E. Person, Elizabeth Clary, Susan Zief, Katie Adamek, Jessica F. Harding

**Affiliations:** 1 Division of Program Development and Operations, Office of Population Affairs, Department of Health and Human Services (HHS), 1101 Wootton Parkway, Suite 200, Rockville, MD 20850 USA; 2 Mathematica, P.O. Box 2393, Princeton, NJ 08543-2393 USA

**Keywords:** Expectant and parenting teens, Young families, High school graduation, Repeat pregnancy, Lessons learned, Teen parents

## Abstract

**Introduction:**

The Pregnancy Assistance Fund (PAF) program funds states and tribes to provide a wide range of services to improve health, social, educational, and economic outcomes for expectant and parenting teens and young adults, their children, and their families. This introductory article to the *Maternal and Child Health Journal* supplement *Supporting Expectant and Parenting Teens: The Pregnancy Assistance Fund* provides a description of the PAF program, including the program goals and structure, participants and communities served, and services provided; presents data on the reach and success of the program; and describes lessons learned from PAF grantees on how to enhance programs and services to have the best outcomes for expectant and parenting young families.

**Methods:**

Performance measure data are used to describe the reach and success of the PAF program, and implementation experiences and lessons learned from PAF grantees were gathered through a standardized review of grantee applications and from interviews with grant administrators.

**Results:**

Since its establishment in 2010, the PAF program has served 109,661 expectant and parenting teens, young adults, and their families across 32 states, including the District of Columbia, and seven tribal organizations; established more than 3400 partnerships; and trained more than 7500 professionals. Expectant and parenting teens and young adults who participated in the PAF program stay in high school, make plans to attend college, and have low rates of repeat pregnancy within a year.

**Conclusions:**

Expectant and parenting teens and young adults in the PAF program demonstrated success in meeting their educational goals and preventing repeat unintended pregnancies. In addition, the staff who implemented the PAF programs learned many lessons for how to enhance programs and services to have the best outcomes for expectant and parenting young families, including creating partnerships to meet the multifaceted needs of teen parents and using evidence-based programs to promote program sustainability.

## Significance

This article presents an overview of the Pregnancy Assistance Fund (PAF) program, a unique program designed to address the multifaceted needs of expectant and parenting teens and young adults, their children, and their families. The article presents data and lessons from the PAF program that suggest that providing expectant and parenting young families with a wide range of coordinated services and supports can result in improved outcomes for the young parents and their children.

## Introduction

Parenting can be difficult at any age, and parenting while still a teenager or a young adult can bring additional challenges. Despite significant reductions in teen births across the United States over the past two decades, 153,152 teens under age 20 and 383,388 young women between ages 20 and 24 became first-time mothers in 2018 (Martin et al. 2019). About one in six (15.8%) births to 15- to 19-year-olds and close to half (47%) of births to 20- to 24-year-olds were to females with one or more children (Martin et al. 2019). Early childbearing can make it more difficult for teens of both genders to finish high school and earn a college degree (Fletcher and Wolfe 2012; Hoffman 2008; Mollborn 2010; Prentice 2012; Shuger 2012). Early childbearing can also increase the likelihood of being poor and experiencing fewer employment opportunities (Hoffman 2008; Bunting 2004). Pregnant teens are also often victims of violence—prior to becoming pregnant, while pregnant, and after giving birth (Futures Without Violence 2010). Experiencing violence during and after pregnancy can increase the likelihood of preterm birth or low birth weight and of having a repeat pregnancy within 24 months (Futures Without Violence 2010; Hill et al. 2016).

To address these challenges, the Pregnancy Assistance Fund (PAF) program aims to improve the health, social, educational, and economic outcomes for expectant and parenting teens and young adults, their children, and their families (Patient Protection and Affordable Care Act 2010). The PAF program is administered by the Office of Population Affairs (OPA; formerly the Office of Adolescent Health) in the U.S. Department of Health and Human Services. Through the PAF program, states and tribes receive funding to provide expectant and parenting young mothers, fathers, and their children with the wide range of services and supports they need to be healthy, complete their education, and secure fulfilling employment.

This article introduces the *Maternal and Child Health Journal* supplement *Supporting Expectant and Parenting Teens: The Pregnancy Assistance Fund* by providing an overview of the PAF program, using performance measure data to describe the reach and success of the PAF program, and sharing implementation experiences and lessons learned from PAF grantees that were gathered through a standardized review of grantee applications and from interviews with grant administrators (Person et al. 2018). This introductory article, along with the other articles in this supplement, describes the diversity of PAF-funded programs across the country and highlights promising practices, strategies, tips, and tools that may be useful to others working to improve outcomes for expectant and parenting young families.

## Overview of the Pregnancy Assistance Fund

**Fig. 1:**
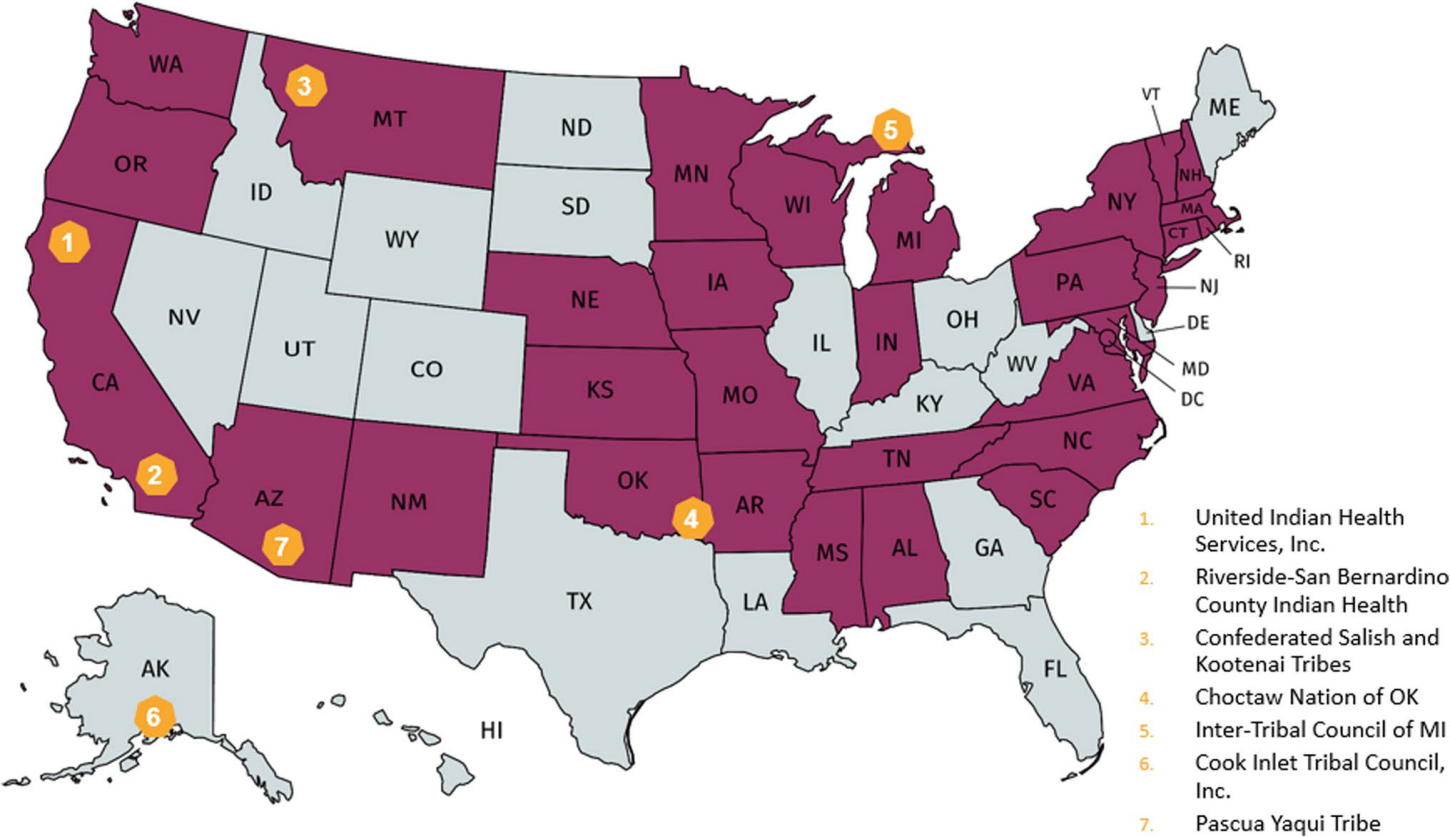
States and tribes funded by the PAF program (2010–2020). *Note* darkly shaded states received PAF funding

The PAF program provides competitive grant funding to states and tribes to improve health, social, educational, and economic outcomes for expectant and parenting young families. To receive grants, states and tribes submit applications to funding opportunity announcements and submissions are reviewed against written criteria by federal staff and an independent review panel. OPA staff then make final award selections to fund states and tribes (referred to here as “PAF grantees”). PAF grantees take different approaches to service delivery, with the majority making subawards to other organizations to provide direct services to young families. States and tribes use PAF funds to establish, maintain, and/or operate expectant and parenting student services in high schools, community service centers, and/or institutions of higher education (IHEs); improve services for pregnant women who are victims of violence; and increase awareness of available services and resources for expectant and parenting young families. The PAF program is unique in its focus on providing access to the wide range of services needed to improve outcomes for expectant and parenting young families. Since 2010, a total of 81 competitive grants have been awarded to 32 states, including the District of Columbia, and seven tribal organizations (Fig. 1) through five distinct cohorts of grants (Table 1). Each cohort of grantees submits a new application for funding, but many states and tribes have been awarded multiple, consecutive grants. Specifically, 13 states and five tribes received a grant for one cohort, 6 states and two tribes received grants for two cohorts, 8 states received a grant for three cohorts, 5 states received grants across four cohorts, no states or tribes received a grant for all five cohorts, and 19 states never received a PAF grant.

**Table 1:** Pregnancy Assistance Fund grant cohorts and service settings (2010–2020)

Grantee cohort	Implementation period	States funded	Tribes funded	Annual funding $	No. participants served	Grantee service setting		
						No. in high schools	No. in community service centers	No. in IHEs
Cohort 1	2010–2013	15	2	500,000–2,000,000	26,442	13	13	3
Cohort 2	2013–2017	17	3	500,000–1,500,000	52,908	15	15	3
Cohort 3	2015–2018	3	0	500,000–800,000	2,723	1	3	0
Cohort 4	2017–2018	15	1	500,000–1,500,000	12,514	8	12	10
Cohort 5	2018–2020	22	3	250,000–1,000,000	15,074	15	24	11
Total	2010–2020				109,661			

A number of states and tribes received grant funding across multiple cohorts. In total, the 81 PAF grants were awarded to 32 unique states, including the District of Columbia, and seven unique tribal organizations. The numbers presented for the service settings are not mutually exclusive because individual grantees can implement in more than one setting. PAF = Pregnancy Assistance Fund; IHE = institution of higher education

The PAF program allows grantees to implement services in high schools, community service centers, and/or IHEs. The choice of where to implement services is up to the funded states and tribes; however, implementing a program in IHEs is unique in that it requires a 25% match on behalf of the IHE. The settings for service delivery have varied over the years of the PAF program (Table 1). In grant cohorts 1, 2, and 3 (n = 40 grantees, with some grantees counted more than once), PAF grantees were asked to select the specific setting or settings where they would provide services. Most grantees selected to provide services in community service centers (31 out of 40 grantees) and high schools (29 out of 40), compared to IHEs (6 out of 40). To expand the reach of PAF services to more expectant and parenting students in IHEs, PAF grantees in cohorts 4 and 5 (n = 41) were asked to provide services across multiple settings, if possible. As a result, the number of grantees providing services in IHEs increased substantially (21 out of 41 grantees) while the number of grantees providing services in community service centers (36 out of 41) and high schools (23 out of 41) remained high. In addition to providing services in community settings, high schools, and IHEs, PAF grantees can also use their funding to improve services for pregnant women who are victims of violence. In grant cohorts 1, 2, and 3 (n = 40), six PAF grantees used their funding to partner with their state attorney general to provide comprehensive violence prevention and intervention services to pregnant women who were victims of violence. Beginning in cohort 4, all PAF grantees were asked to provide violence prevention and intervention services as a part of their PAF grant.

PAF grantees provide access to a wide range of support services to meet the diverse needs of expectant and parenting young families across all implementation settings. Services are either provided directly or through referrals and are focused across five primary areas: (1) personal health (e.g., case management, prenatal care, health insurance enrollment support, behavioral health, violence prevention); (2) child health (e.g., home visiting, access to health care, well-child visits); (3) education and employment (e.g., tutoring, academic support, assistance with college applications, employment and job-readiness training); (4) concrete supports (e.g., food, housing, transportation, baby supplies including diapers, cribs, car seats, etc.); and (5) parenting supports (e.g., parenting and healthy relationship education, child development education, child care). PAF grantees can determine which areas to focus on based on the needs of the population served. For example, in cohorts 2 and 3, the most common focus of PAF programs was on parenting skills (Person et al. 2018). In addition, nearly all PAF grantees implement public awareness and education activities that assist expectant and parenting young families in learning about and accessing available services in their community. Public awareness activities include creating print materials, websites, social media campaigns, and in-person events and participating in public service announcement campaigns like Text4Baby—a free text messaging service that provides subscribers with information on maternal and child health (https://www.acf.hhs.gov/text4baby).

## Key Components of the Pregnancy Assistance Fund

Beyond providing a wide range of supportive services across multiple implementation settings, four additional components of the PAF program are critical to achieving its mission: (1) an emphasis on serving both young mothers and young fathers; (2) the use of a thorough needs and resource assessment to determine what services to provide, where to provide them, and to whom to provide them; (3) reliance on well-coordinated, cross-sectoral partnerships to meet the multifaceted needs of expectant and parenting young families; and (4) the ongoing use of performance measure data to assess program implementation and identify areas for continuous quality improvement.

### Including and Engaging Young Fathers is Critically Important

PAF grantees emphasize serving both young mothers and young fathers. Often, services for expectant and parenting teens and young adults focus on the mother and the baby, and the same emphasis is not placed on engaging and serving young fathers. Given the critical importance of having fathers actively and positively engaged in the lives of their children (Howard et al. 2006), PAF grantees actively recruit, retain, and engage young fathers in all aspects of their projects. Although engaging young fathers has been difficult, PAF grantees have many lessons and promising practices to share about their work to engage young fathers (Niland and Selekman 2020; McGirr et al. forthcoming), and OPA developed a suite of technical assistance products to support grantees and others in their efforts to serve young fathers (OAH 2016).

### A Thorough Needs and Resources Assessment Leads to Robust and Responsive Services

All PAF grantees are required to engage in a thorough needs and resource assessment in each community where services are provided. This assessment determines the needs of the expectant and parenting young families in the community and identifies the resources and services that already exist. The results are used to ensure that services are provided to those persons most in need, tailor services to best meet the needs of participants served, address gaps in services, ensure no duplication of services, and improve access to existing services. Grantees can conduct needs and resource assessments according to their own design, but in their response to the funding opportunity announcements, they must describe how they will assess needs and resources on an ongoing basis to ensure programs continue to be aligned with changing community needs.

### Cross-Sectoral Partnerships Meet the Multifaceted Needs of Expectant and Parenting Young Families

To best address the multifaceted and diverse needs of expectant and parenting young families, PAF grantees establish and maintain numerous partnerships with public and private service providers across sectors (state/tribal/local government, businesses, child care, education, health care, housing, transportation, etc.) in the communities served. For example, some states and tribes required their local providers to develop a collaborative entity (such as a community coalition or local advisory board) as part of their grant. Grantees described that engaging cross-sectoral partners enabled them to provide holistic, wraparound services and ensure coordination across partners, reduce service duplication, and raise awareness and increase the use of available services (Person et al. 2018).

### Collecting and Using Data Strengthens Sound Monitoring and Quality Improvement Practices

Beginning in cohort 2, OPA established a uniform set of performance measures that all PAF grantees collect and report to OPA annually. The performance measures include data on participants served; services and referrals provided; partnerships; trainings; and outcomes focused on high school graduation, enrollment in postsecondary education, and repeat pregnancy. PAF grantees use performance measure data to continuously assess their progress; identify successes, challenges, and lessons learned; and make continuous quality improvements to the services they provide. PAF grantees have used the performance measure data to adjust their recruitment strategies (where and how they recruit participants); their implementation practices (duration and time of parenting classes, referral systems, modality of instruction); and their strategies for raising awareness about available services (social media marketing).

## Reach and Success of the PAF Program

Since 2010, the PAF program has served 109,661 expectant and parenting teens, young adults, and their family members (Table 1); established more than 3400 partnerships; and trained more than 7500 professionals, according to performance data. On average, 54.4% of participants served were expectant and parenting mothers, 8.4% were expectant and parenting fathers, and 37.2% were children. Most expectant and parenting participants were age 18 and older (63.7%), 30.1% were 16–17 years, and 6.2% were age 15 or younger. Most of the PAF participants were white (47.7%) and black or African American (34.9%), and almost half were Hispanic (48%).

On average, across all PAF cohorts, 49% of participants received PAF services in high schools, 38% in community service centers, and 9% in IHEs. In IHEs, 64% of those persons served were enrolled in community colleges, 26% were in four-year colleges or universities, 7% were in vocational or technical schools, and 2% were in other types of IHEs. The majority of participants served in high schools were seniors (29%) and juniors (19%), 12% were sophomores, and 6% were freshman.

The services most commonly provided to participants varied by year. On average, the most common services provided directly or through referrals between 2013 and 2016 were parenting skills, case management, concrete supports, education supports, healthy relationship education, and health care services (Table 2). The services most frequently provided directly by PAF grantees included parenting skills, case management, healthy relationship education, and home visitation services. Services most commonly provided through referrals to partner organizations included education support, concrete supports, health care services, child care, vocational services, and transportation.

**Table 2:** Services most commonly provided through the PAF program between 2013 and 2016

PAF services	Annual average no. of services provided
Parenting skills	7354
Case management	7176
Concrete supports	6323
Education supports	6245
Healthy relationship education	5886
Health care services	5301
Home visitation services	4894
Child care	3306
Vocational services	3238
Transportation	2965

*Note* This question averages the number of services provided annually between 2013 and 2016 because the question was asked consistently in those years

*PAF* Pregnancy Assistance Fund

Federally required performance measure data collected by PAF grantees for fiscal year 2016 show that many expectant and parenting teens and young adults who participated in the PAF program stayed in high school, made plans to attend college, and had low rates of repeat pregnancy within a year (OAH 2017). Grantees collected data elements defined by OPA about the number and types of people served, the types of services provided, and key outcomes and reported them annually to OPA. Although these performance data have limitations, the data elements were defined consistently across grantees and demonstrated positive outcomes for many PAF participants. Pregnancy and parenting contribute to school dropout among teen girls: 30% of female dropouts report these as key reasons they left school (Shuger 2012). However, just 8% of PAF participants who were enrolled in high school dropped out of school during the 2016 reporting year.^1^ Furthermore, although only 5% of teens who became mothers before age 17 complete two years of college by their late 20s (Hoffman 2006), 52% of PAF program participants who were high school seniors or those participants enrolled in GED programs were accepted into an IHE during the 2016 reporting year.^2^ Finally, although 17% of births to teens nationally are a repeat birth (Martin et al. 2019), PAF programs reported that only 6% of their participants had another pregnancy during the 2016 reporting year.^3^

In addition to documenting suggestive findings from grantee performance measure data, OPA contracted with Mathematica to conduct a rigorous impact study of New Heights, a PAF-funded project in Washington, D.C. New Heights is a school-based program that places a dedicated coordinator in each school who is responsible for providing advocacy services, targeted case management, weekly educational workshops, and concrete supports to the expectant and parenting students within the school. The evaluation found that New Heights increased school attendance and credits earned, reduced unexcused absences, and increased the graduation rate among the expectant and parenting teens and young adults who received the program (Asheer et al. 2017a). An article that describes the unique evaluation design, which relied on low-cost administrative data, is included in this supplement (Zief et al. forthcoming). OPA also contracted with Mathematica to conduct a randomized controlled trial of Healthy Families Healthy Futures enhanced with Steps to Success, a home visiting program for teen parents in Houston focused on improving parenting skills, preventing abuse, and reducing rapid repeat pregnancies. The evaluation found a statistically significant 20.8 percentage point increase in exposure to information on parenting and a 15.4 percentage point increase in exposure to information on methods of birth control as well as suggestive evidence of an 11 percentage point increase in use of long-acting reversible contraceptives and an 8.9 percentage point decrease in knowledge about birth control pills (Zief et al. forthcoming). An evaluation of the California-funded PAF project is also currently underway, with findings from its implementation reported in this supplement (Asheer et al. forthcoming).

## Insights and Lessons from Implementing PAF Programs

Beginning in 2013, OAH conducted interviews with PAF grantees to document their implementation experiences and lessons learned (Person et al. 2018). Program challenges and lessons learned from how grantees addressed them are included below and provide important insights for others interested in serving expectant and parenting young families.

**Establishing and maintaining diverse partnerships is key for addressing the wide range of services needed to improve health, social, educational, and economic outcomes for young families.** Grantees leveraged both formal and informal partnerships to reduce barriers to accessing services, link participants with specific services, and support program sustainability. PAF grantees stressed the importance of working with partners across different sectors (i.e., health, education, and social services) to be able to provide expectant and parenting young families with the full range of services they need.

**Difficulty coordinating services across many existing providers made it challenging for expectant and parenting young families to receive the full range of services they needed.** Program administrators reported that prior to receiving PAF funding, expectant and parenting youth were not well served in their communities, but not because of a lack of available resources. Rather, the problem was a lack of services specifically for the expectant and parenting teen and young adult population; a lack of knowledge of and access to available programs and services; and a lack of coordination among service providers that prevented youth from getting the holistic, wraparound services they needed. Therefore, PAF funding was used to fill service gaps, enhance existing programs, and improve coordination. PAF grantees used their PAF funding to improve services for expectant and parenting young families by (1) providing new services or targeting specific underserved subpopulations to fill gaps in existing services; (2) enhancing existing programs by adding or refining services while also expanding programs to serve more youth; and (3) improving coordination across state or tribal agencies and among local program providers to make holistic, wraparound services more readily accessible to expectant and parenting young families, avoid the duplication of services, and support the sustainability of PAF programs.

**Grantees perceived a lack of evidence-based programs to address the unique needs of expectant and parenting young families.** Nearly all grantees tried to find evidence-based or evidence-informed programs that had been shown to improve health, social, educational, and economic outcomes but expressed that they experienced challenges finding programs to meet the specific needs of expectant and parenting young families. One challenge was a lack of evidence for broader, more collaborative approaches used to address the multifaceted needs of expectant and parenting young families. Another challenge was that grantees perceived that many of the most common evidence-based programs designed to addresses relevant maternal and child health outcomes (e.g., home visiting programs, substance abuse programs, parenting programs) were not designed specifically for teens and young adults. For example, some home visiting programs require participation start early in the pregnancy, before some teens recognize their needs and seek services. Some were also too limited in focus and did not adequately address areas of importance for young families, including educational goals and access to services. Nonetheless, many grantees used evidence-based programs, such as the Parents as Teachers home visiting curriculum and Nurturing Parenting curriculum for preventing and treating child abuse and neglect. Using evidence-based programs was helpful for supporting sustainability because this could enhance grantees’ eligibility for additional funding streams, such as the Maternal, Infant, and Early Childhood Home Visiting Program (Asheer et al. 2017b).

## Conclusion

The PAF program takes a comprehensive approach to addressing the needs of expectant and parenting young families. PAF grantees implemented programs across multiple settings including high schools, community service centers, and IHEs; provided a wide range of coordinated services to mothers, fathers, and their children; and served pregnant women who are victims of violence. Since its establishment in 2010, the PAF program has served 109,661 expectant and parenting teens, young adults, and their family members across 32 states, including the District of Columbia, and seven tribal organizations. Expectant and parenting teens and young adults in the PAF program demonstrated success in meeting their educational goals and preventing repeat unintended pregnancies. In addition, the staff who implemented the PAF programs learned many lessons for how to enhance programs and services to have the best outcomes for expectant and parenting young families, including creating partnerships to meet the multifaceted needs of teen parents and using evidence-based programs to promote program sustainability.

## Footnotes

1 The denominator (number of students in high school) underestimates the total number of participants at risk of dropping out because some grantees reported the number of students in high school at the end of the year and omitted participants who dropped out during the course of the year.

2 The denominator (number of participants who were either seniors or preparing for a GED) underestimates the total number of participants eligible to be accepted into an IHE because some grantees reported the number of participants who were seniors or preparing for a GED at the end of the year and omitted participants who had already graduated or completed the GED earlier in the year.

3 The denominator (parenting teen mothers) underestimates the total number of participants at risk for a repeat pregnancy because some grantees counted participants as parenting or expectant based on their status at the beginning of their program participation; expectant teens who delivered during the course of the year would thus be excluded from the count of teens at risk of a repeat pregnancy.
